# Diagnostic difficulties and pitfalls in rapid on-site evaluation of endobronchial ultrasound guided fine needle aspiration

**DOI:** 10.4103/1742-6413.64385

**Published:** 2010-06-14

**Authors:** Sara E. Monaco*, Matthew J. Schuchert, Walid E. Khalbuss

**Affiliations:** Department of Pathology, University of Pittsburgh Medical Center, Pittsburgh, Pennsylvania; 1 Department of Thoracic Surgery, University of Pittsburgh Medical Center, Pittsburgh, Pennsylvania

**Keywords:** Cytopathology, EBUS, endobronchial ultrasound, fine needle aspiration

## Abstract

**Background:**

One of the novel techniques utilizing fine needle aspiration (FNA) in the diagnosis of mediastinal and lung lesions is the endobronchial ultrasound (EBUS)-guided FNA. In this study, we describe five cases which had a discrepancy between on-site evaluation and final diagnosis, or a diagnostic dilemma when rendering the preliminary diagnosis, in order to illustrate some of the diagnostic difficulties and pitfalls that can occur in EBUS FNA.

**Methods:**

A total of five EBUS FNA cases from five patients were identified in our records with a discrepancy between the rapid on-site evaluation (ROSE) and final diagnosis, or that addressed a diagnostic dilemma. All of the cases had histological confirmation or follow-up. The cytomorphology in the direct smears, cell block, and immunohistochemical stains were reviewed, along with the clinical history and other available information.

**Results:**

Two cases were identified with a nondefinitive diagnosis at ROSE that were later diagnosed as malignant (metastatic signet-ring cell adenocarcinoma and metastatic renal cell carcinoma (RCC)) on the final cytological diagnosis. Three additional cases were identified with a ROSE and final diagnosis of malignant (large cell neuroendocrine carcinoma (LCNEC) and two squamous cell carcinomas), but raised important diagnostic dilemmas. These cases highlight the importance of recognizing discohesive malignant cells and bland neoplasms on EBUS FNA, which may lead to a negative or a nondefinitive preliminary diagnosis. Neuroendocrine tumors can also be difficult due to the wide range of entities in the differential diagnosis, including benign lymphocytes, lymphomas, small and nonsmall cell carcinomas, and the lack of immunohistochemical stains at the time of ROSE. Finally, the background material in EBUS FNAs may be misleading and unrelated to the cells of interest.

**Conclusions:**

This study illustrates the cytomorphology of five EBUS FNA cases that address some of the diagnostic challenges witnessed while examining these specimens during ROSE. Many of the difficulties faced can be attributed to the baseline cellularity of the aspirates, the bronchial contamination, the difficulty identifying neoplasms with bland cytology, the wide spectrum of diseases that can occur in the mediastinum with overlapping cytomorphologic features, the mismatch between the background material and the cell populations present, and the overall unfamiliarity with these types of specimens.

## INTRODUCTION

Given the spectrum of lesions that can occur in the mediastinum and the false positive results that can occur with radiologic imaging, obtaining a pathologic diagnosis for mediastinal lesions is crucial for staging and planning the appropriate treatment.[1‐4] The traditional diagnostic modalities have included noninvasive or minimally invasive approaches utilizing exfoliative cytology (sputum, bronchial brushing, bronchial washing, pleural fluid) and aspiration cytology [transbronchial needle aspiration or Wang needle, CT-guided Fine needle aspiration (FNA)], in addition to more invasive approaches utilizing surgical pathology (mediastinoscopy, thoracoscopy).

Endobronchial ultrasound (EBUS)-guided FNA has emerged as a novel technique utilizing aspiration cytology to obtain a pathologic diagnosis of mediastinal and lung lesions.[5‐10] This technique is particularly appealing as it is minimally invasive, safe, and utilizes real-time ultrasound guidance. The image guidance feature distinguishes it from other traditional methods, such as the transbronchial FNA or Wang needle biopsy, and increases the diagnostic yield of the procedure by allowing for direct visualization of the needle in the target, while also decreasing the possibility of complications.[11,12] In addition, EBUS FNA allows for a broader access to lymph nodes in the mediastinum than mediastinoscopy.[9] EBUS FNA is also advantageous in scenarios where more invasive procedures are contraindicated or unnecessary, such as patients who are poor surgical candidates, patients suspected of having nonsurgical diseases (lymphoma, germ cell tumors), and for restaging procedures in patients with postprocedural fibrosis from prior surgeries.

Rapid on-site evaluation (ROSE) is an important aspect of EBUS FNA, as it frequently determines whether a patient will go on for a more invasive procedure, such as mediastinoscopy.[10] The main issues that surgeons or clinicians expect the cytopathologist to address in the ROSE are: whether the specimen is adequate, whether the lesion is benign or malignant, and whether there is sufficient material for a definitive final diagnosis or subclassification. However, these determinations can be difficult due to the lack of standardized adequacy criteria, the wide variety of mediastinal/lung lesions to consider, the contamination of the needle, the lack of ancillary studies during ROSE, and the unfamiliarity with these specimens. In addition, the baseline cellularity and background material in these specimens, in part due to the bronchial contamination, give rise to diagnostic difficulties and pitfalls which have only been described in a few recent publications.[10,13,14]

Although some of the cytomorphologic pitfalls in pulmonary and mediastinal lesions have been described previously, [15‐17] the diagnostic difficulties unique to EBUS, with a focus on the dilemmas seen at ROSE, are not well-characterized. The aim of this study was to review some of the diagnostic difficulties and pitfalls that we have seen in our initial years of experience with EBUS FNA during ROSE, and to highlight the cytopathology issues to be aware of when evaluating these specimens.

## MATERIALS AND METHODS

During a period of forty-six months between September 2006 and October 2009, 491 EBUS-guided FNAs were performed at the University of Pittsburgh Medical Center and were reviewed. The cases included in this study were identified through cytology-histology correlations and interesting cases identified through retrospective review that address a diagnostic difficulty. Two cases illustrate a discrepancy between the ROSE diagnosis and the final diagnosis. In addition, three other unusual or difficult cases were identified which addressed a diagnostic difficulty at the time of ROSE. Each case had histologic and ancillary study confirmation. The cytomorphology and ancillary studies, such as immunohistochemical studies (IHC), in addition to the available clinical information, and histologic material were retrospectively reviewed. This study was reviewed and approved by the institutional review board at the University of Pittsburgh Medical Center (IRB#PRO10040040).

### Cytomorphological assessment

The EBUS FNAs were either performed by a pulmonologist in the bronchoscopy suite or by a thoracic surgeon in the operating room. Each case had ROSE by a cytopathologist for adequacy assessment and interpretation, which allowed appropriate triage of the specimen and directed patient care. The ROSE by the cytopathologist involved examination of an air-dried Diff-Quik^TM^ (DQ stain; Protocol Hema 3, Fisher Scientific, Kalamazoo, MI) stained smear from each FNA pass. Additional slides were fixed in 95% alcohol for Papanicolaou staining. The remaining material was fixed in formalin for a cell block preparation. The number of FNA passes varied based on the ability to obtain adequate material and the ability of the procedure to be tolerated, but in general, approximately 3-5 passes were used.

At the time of final interpretation, the Papanicolaou and DQ-stained smears, in addition to the hematoxylin and eosin (H&E) stained sections of the formalin-fixed cell block and any accompanying surgical biopsies obtained, were evaluated and interpreted in conjunction with the results from ancillary studies and prior available pathology in order to render a final cytology diagnosis. The cytomorphologic findings in each case are discussed along with the pitfalls and differential diagnoses to consider.

## RESULTS AND DISCUSSION

### Case 1: A case of dyscohesive single cells

**Figure 1:**
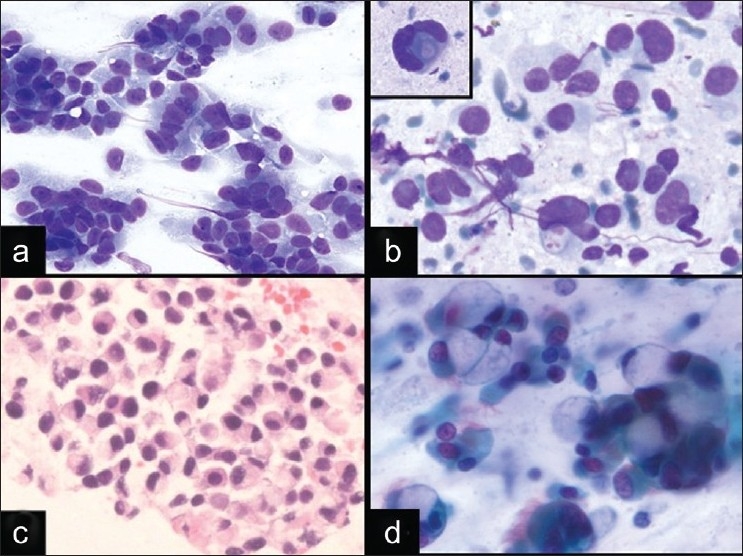
Signet-ring adenocarcinoma in EBUS FNA and diagnostic pitfall. a, b) An EBUS FNA of demonstrates loosely cohesive tumor cells with relatively homogeneous nuclei, variable amounts of glassy cytoplasm, and some plasmacytoid cells (a. DQ stain, ×400; B. DQ stain, ×600). Occasional cells have marked nuclear enlargement, prominent nucleoli, and cytoplasmic vacuolization (b inset. DQ stain, ×600). c) Cell block from signet-ring adenocarcinoma (HandE stain, ×400). d) Goblet cell metaplasia from bronchial contamination (Pap stain, ×600) can mimic signet-ring adenocarcinomas.

A 52-year-old woman with a history of poorly differentiated adenocarcinoma of the stomach three years prior to presentation, was found to have a 2.0 cm, fluorodeoxyglucose (FDG) avid right paratracheal lymph node on a CT/PET scan. EBUS FNA was performed and revealed discohesive cells with mild nuclear pleomorphism and variable amounts of cytoplasm. The nuclei were enlarged, round, and hyperchromatic with prominent nucleoli [Figure 1a and 1b]. Some cells appeared as naked nuclei, while others had eccentrically placed cytoplasm and scattered cytoplasmic vacuoles with an occasional targetoid appearance [Figure 1b]. The background contained reactive bronchial epithelial cells and a few crushed lymphoid cells. The ROSE rendered was atypical due to the uncertainty of whether the atypical cells represented metastatic adenocarcinoma or reactive bronchial epithelial cells with goblet cell metaplasia. Immunohistochemical and special stains performed on the cell block [Figure 1c] confirmed that the cells were positive for CDX2 and mucicarmine, while negative for TTF-1. The final cytological diagnosis was metastatic adenocarcinoma of gastrointestinal origin with signet-ring features, which was similar in morphology to the patient’s primary gastric tumor, which also had involvement of four lymph nodes at the time of the original gastrectomy.

Signet-ring adenocarcinomas are notoriously difficult in aspiration cytology, due to the discohesive nature and sometimes bland atypia, which make their distinction from cells with reactive atypia difficult. In EBUS FNA, these tumor cells can be difficult to distinguish from bronchial cells at low power due to their round nuclei and moderate amounts of eccentrically placed cytoplasm, which can sometimes impart a vague columnar-look to the cells. Signet-ring adenocarcinomas can also mimic histiocytes due to the vacuolated cytoplasm and discohesion. Goblet cell metaplasia also has overlapping cytomorphologic features in that the bronchial cells can have large cytoplasmic vacuoles imparting a “signet-ring”-like appearance [Figure 1d], as previously described in the literature.[18,19] Although signet-ring type adenocarcinomas of the lung are rare, adenocarcinomas with signet-ring morphology can arise from a variety of locations. The most common location is the stomach or gastroesophageal junction, and these tumors are characterized by their poorly differentiated nature and aggressive behavior with diffuse spread.[20] In addition, signet-ring changes have been described in tumors other than adenocarcinoma, which may occur in the mediastinum, such as plasmacytomas,[21] melanomas,[22] mesotheliomas,[23,24] seminomas,[25] schwannomas,[26] and others, which underscores the importance of clinical history during ROSE and the need for cell block material for ancillary studies.

Accurate identification of signet-ring adenocarcinomas in EBUS FNA relies on very careful examination of the cells present to look for malignant cells with nuclear enlargement, prominent nucleoli, and irregular nuclear contours, particularly because immunohistochemical stains are not available at the time of ROSE. At low power, some cells may have a resemblance to reactive bronchial cells; however, the nuclear atypia and nuclear enlargement in comparison to nearby red blood cells, in addition to the lack of cilia in signet-ring adenocarcinomas, can help in differentiating the tumor cells from reactive bronchial cells. The features that help to distinguish these cells from histiocytes include the presence of large mucin vacuoles and lack of anthracotic pigment. Goblet cell metaplasia differs in that it usually lacks nuclear atypia, occurs in cohesive clusters, and is seen intermixed with ciliated bronchial epithelial cells [Figure 1d]. [19] In addition to these cytomorphologic findings, an appropriate clinical history of a prior signet-ring cell tumor, when available, can be very helpful in these cases.

### Case 2: Pitfalls of granulomas and bland cytomorphology

**Figure 2:**
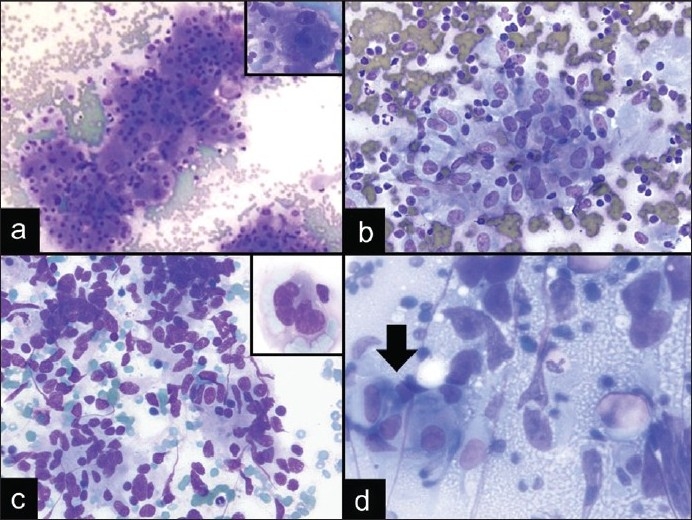
Diagnostic pitfalls of granulomatous inflammation in EBUS FNA. a) Metastatic RCC with occasional cells showing nuclear enlargement and large prominent nucleoli (DQ stain, ×200; inset: DQ stain, ×600). b) Nonnecrotizing granulomatous inflammation in a patient with sarcoidosis (DQ stain, ×400). c) Spindle cell melanoma with spindle-shaped nuclei that resemble epithelioid histiocytes (DQ stain, ×400; inset: DQ stain, ×600). d) Malignancies, such as mediastinal germ cell tumors, can also be associated with granulomas (DQ stain, ×600; arrow indicates granuloma)

A 60-year-old female smoker with a remote history of renal cell carcinoma (RCC) presented with an enlarged right paratracheal lymph node. The clinical differential was reactive lymphoid hyperplasia, granulomatous inflammation, or metastatic carcinoma from the lung or kidney. The DQ-stained smears from the EBUS FNA showed irregular clusters of cells with abundant cytoplasm containing some vacuolization and round-to-oval nuclei with occasional nucleoli [Figure 2a]. In addition, some clusters appeared to be associated with transversing small blood vessels. Upon closer examination, a few cells with nuclear pleomorphism were noted, including cells with large prominent nucleoli and binucleation [Figure 2a inset]. The ROSE diagnosis was atypical and the diagnosis was deferred due to the atypical epithelioid cells present, which were felt to either represent nonnecrotizing granulomas or metastatic tumor. Additional material was submitted for cell block preparation. The immunohistochemical stains confirmed that the cells were positive for cytokeratin, vimentin, CD10, and EMA. The cells stained negative for CD68. Thus, the final cytologic diagnosis was metastatic RCC, which was confirmed on follow-up surgical excision of the lymph node.

This case illustrates the diagnostic difficulty, particularly at the time of ROSE, in differentiating cytologically bland neoplasms from the reactive atypia of epithelioid histiocytes in granulomatous inflammation, as illustrated in a case of sarcoidosis [Figure 2b]. This diagnostic difficulty was highlighted in a published study looking at the intraoperative cytologic interpretation in 156 cases of granulomatous inflammation, in which 9 cases (6%) showed a clinically significant discrepancy where a neoplasm was misdiagnosed as a granuloma or vice versa. [27]

These discrepancies occur due to the variable features of granulomas and the cytologic atypia seen in epithelioid histiocytes, which can include prominent nucleoli and nuclear enlargement [Figure 2b]. Also, the necrotic background appreciated in necrotizing granulomas can lead one to make a false positive interpretation of a neoplastic process. The cytomorphology of epithelioid histiocytes within granulomatous inflammation can also resemble sinus histiocytosis or other histiocytic processes in the mediastinum,[28] spindle cells in spindle cell lesions, such as seen in a case of a spindle cell melanoma [Figure 2c], and germinal center cells or proliferative vascular endothelial/smooth muscle cells.[14] An additional pitfall is that granulomas can be associated with a wide spectrum of entities, including benign or infectious processes and neoplastic processes, such as seminomas [Figure 2d], lymphomas, and metastatic carcinomas. So even if granulomatous inflammation is the predominant cytomorphologic feature of the aspirates, one should still search thoroughly for atypical cells indicative of a malignancy. In difficult cases, immunohistochemical stains can help, in that histiocytes are CD68 positive, while other tumors in the differential will usually be CD68 negative, and have their characteristic immunoprofile, as seen in this case of metastatic RCC. This case illustrates that the distinction of granulomatous inflammation from its mimics is sometimes challenging during ROSE; however, clinical history, careful examination of the cytomorphology, and awareness of pitfalls can help in accurate identification.

In addition to the pitfalls of recognizing granulomas, low grade RCC can be particularly difficult to diagnose in cytological specimens due to the low nuclear-to-cytoplasmic ratios of the tumor cells, abundant cytoplasm, and sometimes bland nuclear pleomorphism, which make its distinction from oncocytic lesions and reactive conditions difficult in some cases. [29] For this reason, in a subset of cases, adequate material for confirmatory immunohistochemical stains is crucial in order to reach a definitive diagnosis. [30] In one institution’s experience, it was reported that approximately 6% of cases had a discrepancy between the preliminary and final diagnosis, and this most commonly involved cases with a change from “nondiagnostic” or “benign” at ROSE to malignant at final diagnosis, which was partially attributed to the additional material available at the time of final diagnosis.[31] Given that cell block and immunohistochemical stains are not available at the time of ROSE, examination of the cytomorphologic features is crucial. In this case, recognition of the scattered cells with large nucleoli and marked nuclear enlargement, in addition to the more well-defined cytoplasmic borders and clusters with transversing vasculature, may have led to a more definitive diagnosis in this case and potentially avoided the need for mediastinoscopy.

### Case 3: Pitfalls of neuroendocrine cytomorphology

**Figure 3:**
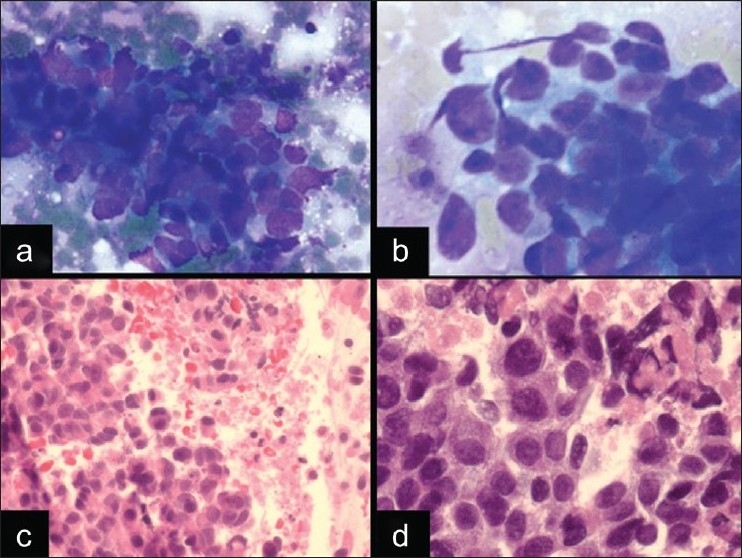
A case of LCNEC in EBUS FNA. a, b) The cytomorphology shows loosely cohesive clusters of cells with nuclear molding, hyperchromasia, and crush artifact, which mimics a small cell carcinoma (a. DQ stain, ×400). However, there are also cells with more abundant cytoplasm and prominent nucleoli (b. DQ stain, ×600). c,d) The cell block showed areas of necrosis and highlights the eosinophilic cytoplasm of the tumor cells, which is most consistent with a LCNEC [(H and E stain, ×400 (C) and ×600 (D)]

A 60-year-old man with a long history of smoking presented with a 4 cm hilar mass of the left lung and no prior history of malignancy. EBUS FNA and transbronchial biopsy were performed of the hilar mass. The DQ-stained smears demonstrated cohesive and discohesive cells with marked crush artifact and nuclear distortion; however, in the areas that appeared better preserved, the nuclei appeared to show nuclear molding [Figure 3a]. Although some of the cells had scant cytoplasm and inconspicuous nucleoli; there were also cells with noticeable amounts of cytoplasm, enlarged nuclei, and nucleoli [Figure 3b-d]. The background was necrotic. Given these findings, the ROSE was malignant, and a small cell lung carcinoma (SCLC) was favored. Immunohistochemical stains performed on the cell block confirmed the neuroendocrine differentiation of the tumor (positivity for CD56 and synaptophysin), the high Ki67 proliferation index (approximately 60%), and demonstrated positivity for TTF-1. The tumor cells on the cell block showed more abundant eosinophilic cytoplasm and prominent nucleoli [Figure 3c-d], similar to that seen in the transbronchial biopsy, which was felt to be compatible with a large cell neuroendocrine carcinoma (LCNEC). The final diagnosis of both the EBUS FNA and transbronchial biopsy was high-grade neuroendocrine carcinoma, most compatible with LCNEC.

**Figure 4:**
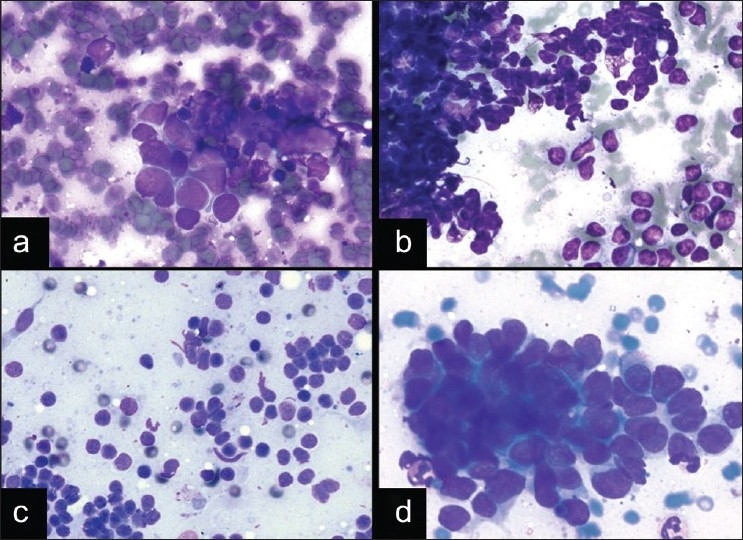
Neuroendocrine carcinomas and pitfalls in EBUS FNA. a) Small cell carcinoma showing nuclear molding, scant cytoplasm, inconspicuous nucleoli, and an apoptotic background (DQ stain, ×400). b) Lymphoma showing pseudo-epithelioid clustering, crush artifact, and a background of lymphoglandular bodies (DQ stain, ×200). c) Benign lymphocytes of a reactive lymph node in EBUS (DQ stain, ×200). d) Basaloid squamous cell carcinoma, which is a nonsmall cell carcinoma that can mimic neuroendocrine carcinomas (DQ stain, ×400)

The correct classification of a neuroendocrine tumor as a SCLC or a LCNEC is a particularly difficult dilemma with which cytopathologists struggle due to the the overlapping features. Both tumors can show nuclear molding, crush artifact, mitoses, necrosis, and variably prominent nucleoli.[32] Although most SCLCs are distinguished by their smaller nuclei with inconspicuous nucleoli, scant cytoplasm and more prominent nuclear molding [Figure 4a], some SCLCs can show variably prominent nucleoli and moderate amounts of cytoplasm making the distinction difficult. In the diagnosis of primary lung lesions, this distinction is important because of the different treatment strategies whereby LCNEC is considered a nonsmall cell carcinoma treated with surgical excision, whereas SCLC is treated with chemotherapy. Although LCNECs appear to have a worse prognosis when compared to other non-small cell carcinomas,[35‐35] the biological behavior of these tumors is not entirely clear, given the rarity of cases, the limited numbers of cases in most published studies, and the difficulty recognizing this type of tumor with about 50% cases misclassified.[36] However, in one of the largest series of LCNECs, patients with this diagnosis appeared to respond best to the chemotherapy regimen used in SCLC.[36] Thus, the distinction of SCLC and LCNEC may not be as clinically important in the metastatic setting. Therefore, simply recognizing a high-grade neuroendocrine carcinoma may be sufficient in EBUS FNA for the appropriate treatment, and thus, definitively deciding whether it is a SCLC or a LCNEC may not be as crucial in metastases.

The differential diagnosis for neuroendocrine tumors by cytomorphology also includes lymphoma [Figure 4b] and benign lymphocytes [Figure 4c]. Given the large number of background lymphocytes in EBUS FNA if the targeted lymph node is sampled, the distinction between lymphoid cells and a neuroendocrine tumor, like SCLC, can be particularly difficult. Both can show crush artifact, discohesive cells, and small hyperchromatic nuclei with scant cytoplasm. However, SCLCs usually have at least a few clusters with evidence of molding, some nuclear enlargement, a necrotic background, and no lymphoglandular bodies. However, in some lymph nodes involved by SCLC, there may be lymphoglandular bodies from the background lymphoid cells, which makes this distinction particularly difficult in EBUS FNA, in comparison to CT-guided FNAs of the lung for primary SCLCs. Another problem that can occur in EBUS FNA is the issue of poorly preserved specimens with crush artifact and nuclear distortion, due to the fragility of lymphoid cells and tumor cells of a SCLC. Therefore, in some cases, a definitive malignant diagnosis may not be possible at the time of ROSE.

In addition to LCNEC, another nonsmall cell carcinoma that can mimic SCLC is basaloid carcinoma, which is usually a variant of squamous cell carcinoma [Figure 4d]. The overlapping cytomorphologic features include cells with scant cytoplasm and nuclear molding; however, these tumors are usually distinguished based on the large cohesive clusters with palisading of the nuclei, the dense cytoplasm, and the presence of spindle cells.[37,38] Immunohistochemical stains performed on the cell block can be very helpful in the differential diagnosis of basaloid carcinoma and SCLC, in that basaloid carcinomas are usually negative for TTF1, and positive for 34βE12 (CK903) and p63. However, SCLCs are usually more diffusely positive for neuroendocrine markers (CD56 and synaptophysin) and TTF1.[39] Furthermore, SCLCs can also be difficult to distinguish from carcinoid tumors, particularly atypical carcinoids, which can have a necrotic background.[40] Carcinoid tumors or well-to-moderately differentiated neuroendocrine tumors usually have relatively monomorphic, round nuclei and the cells can be seen in small rosettes or associated with thin-walled blood vessels.[40,41] If a cell block is available at the time of final diagnosis, a Ki67 proliferation index can be helpful, in that carcinoid tumors usually have a lower proliferation index (typically <20%), compared to SCLCs with a high Ki67 proliferation index (>50%).[40]

Table 1 summarizes some of the cytomorphologic features that can help in distinguishing the entities in the differential diagnosis of a LCNEC.

**Table 1:** Comparison of cytomorphologic features in neuroendocrine tumors and mimics

	*Lymphocytes/Lymphoma*	*Carcinoid*	*SCLC*	*LCNEC*	*Basaloid carcinoma*
Cell arrangement	Discohesive, Pseudo-epithelioid clusters in some	Discohesive and Loosely cohesive; rosettes; associated with blood vessels	Cohesive and discohesive	Cohesive	Cohesive ± nuclear palisading at the edge of groups
Nuclei	Small, round, coarse chromatin	Small, round, monomorphism, stippled chromatin; no molding	Small, molding, pleomorphism, stippled chromatin	Larger, occasional molding	Coarse chromatin, oval/spindle cell nuclei
Nucleoli	May be prominent in immature cells/lymphoma	Inconspicuous	Inconspicuous, occasional nucleoli in “intermediate” type	Prominent	Usually absent
Cytoplasm	Scant/Absent, Basophilic	More abundant, granular, “plasmacytoid”	Scant/ Absent	More abundant, granular	More abundant, Dense
Background	Lymphoglandular bodies	Branching capillaries; Occasionally necrotic (atypical carcinoid)	Apoptotic and Necrotic	Apoptotic and Necrotic	Sometimes necrotic

### Case 4: Pitfalls of mucinous background material

**Figure 5:**
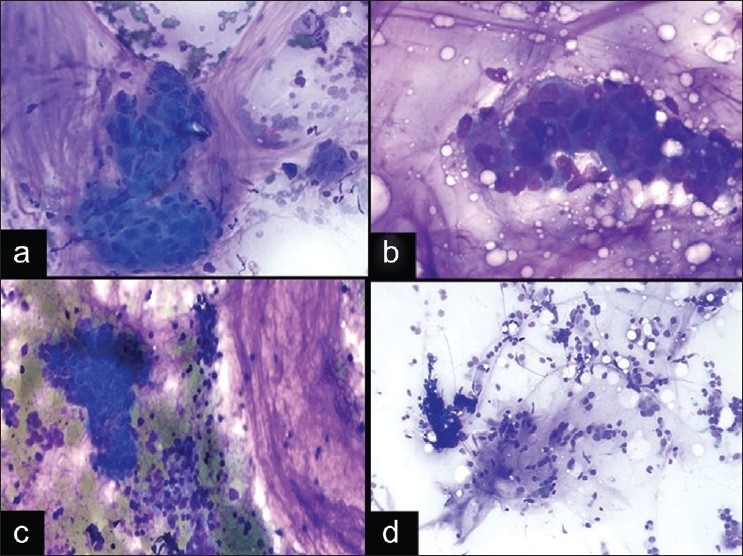
Pitfalls of a mucinous background. a) Squamous cell carcinoma showing clusters of cells with nuclear pleomorphism and dense cytoplasm in a background of mucinous material with histiocytes, inflammatory cells, and bronchial cells from bronchial contamination (DQ stain, ×200). b) Mucinous adenocarcinoma with tumor cells floating in a background of mucinous material without inflammatory cells or macrophages (DQ stain, ×400). c) Small cell carcinoma with a mucinous background (DQ stain, ×200). d) Nondiagnostic EBUS FNA with bronchial contamination (DQ stain, ×200)

A 49-year-old male smoker with a history of squamous cell carcinoma was found to have a 4 cm left hilar mass on CT scan. An EBUS FNA performed of the mass demonstrated abundant mucinous material with clusters of pleomorphic cells [Figure 5a]. The nuclei were enlarged and hyperchromatic, with coarse chromatin and dense basophilic cytoplasm. The background showed abundant mucinous material with entrapped inflammatory cells and bronchial cells [Figure 5a]. The ROSE was malignant, favoring a nonsmall cell carcinoma; however, it was uncertain if the mucin was associated with the neoplastic cells or simply a contaminant. The immunohistochemical stains performed on sections of the cell block revealed that the tumor cells were positive for p63, CK5/6, and negative for TTF1. In addition, the tumor was similar in morphology to the patient’s prior lung biopsy with a diagnosis of squamous cell carcinoma. The final diagnosis was metastatic squamous cell carcinoma with the mucin attributed to bronchial contamination.

This case illustrates the discrepancy or mismatch that may exist between background material and the cells of interest in EBUS FNA. For example, a mucinous background usually leads us to infer that there is a mucin-producing adenocarcinoma [Figure 5b]; however, similar to pancreatic EUS-guided FNAs, mucin can be a contaminant from the needle path and is not necessarily produced by the tumor cells present. This is an important aspect of EBUS FNA to be aware of, particularly because cytopathologists often rely on the background material to aid in making a diagnosis and for subtyping tumors.

When looking at an EBUS FNA with a mucinous background, there are two cytomorphologic clues that can help to determine if the mucin is truly indicative of a mucin-producing adenocarcinoma. The first clue is the presence of columnar cells, or cells with voluminous mucinous cytoplasm or targetoid vacuoles with a central mucin droplet. A second clue is the presence of “dirty” mucin with entrapped inflammatory cells and debris, which usually signifies mucin contamination [Figure 5a, 5c and 5d]. This is in contrast to mucinous adenocarcinomas [Figure 5b], which usually have a “clean” mucinous background with thick mucin that does not have entrapped inflammatory cells or debris. In our experience with EBUS FNA, we have seen several tumors, including small cell carcinoma [Figure 5c] and this case of squamous cell carcinoma [Figure 5a], associated with mucinous contamination. Other cystic mucinous neoplasms occurring in the lung or mediastinum should also be considered, such as mucinous cystadenocarcinoma, which has been described in the lung.[42] The final thing to consider is mucinous contamination only, without associated tumor cells or lymphocytes [Figure 5d], which indicates a nondiagnostic specimen and the need to ask the surgeon for additional material. In some cases, the presence of reactive bronchial cells with cytologic atypia within a mucinous background, may present a diagnostic difficulty in determining whether the specimen is simply bronchial contamination (and therefore nondiagnostic) or truly diagnostic of a metastatic mucinous adenocarcinoma.

In addition to a mucinous background, other types of background material are associated with a particular differential diagnosis in EBUS FNAs, and the four main categories of background material with their associations are summarized in Table 2.

**Table 2:** Differential diagnosis based on background material

*Necrotic background*	*Mucinous background*	*Tigroid background*	Lymphoid background
Necrotizing granulomas	Nondiagnostic with mucin contamination	Germ cell tumor/Seminoma	Adequate, Negative for malignant cells with lymph node sampling
High grade tumor with necrosis	Negative for malignant cells with mucin contamination (lymphocytes present)	Glycogenated neoplasm (Squamous cell carcinoma, Ewing’s Sarcoma, clear cell tumors, others)	Lymphoma
	Mucinous adenocarcinoma		Mimics seen with small round blue cell tumors (example: necrotic/apoptotic background of small cell carcinoma)
	Mucinous Cystic Neoplasms [33] Malignant neoplasm with mucin contamination		

### Case 5: Pitfalls of tigroid background material

**Figure 6:**
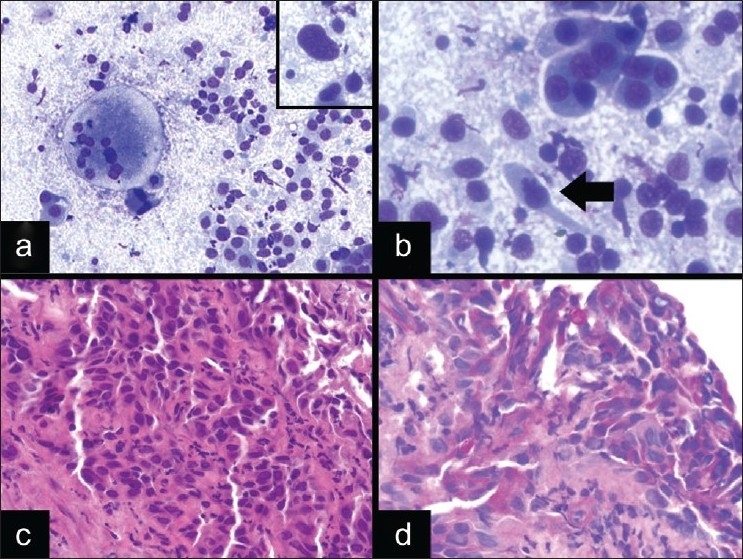
Squamous cell carcinoma with a tigroid background. a) There are discohesive cells with nuclear enlargement and pleomorphism, in a tigroid-like background with multinucleated giant cells (DQ stain, ×200; A inset, ×600). b) The tumor cells have dense cytoplasm and occasional tadpole cells (arrow) are also seen (DQ stain, ×600). c) The corresponding lung biopsy showed an invasive poorly differentiated squamous cell carcinoma (H and E stain, ×400). d) PAS stain performed on the biopsy highlighted the glycogen within the tumor cells, and was digested with diastase (PAS stain, ×400)

A 69-year-old woman, who presented with hemoptysis and no prior history of malignancy, was found to have an FDG-avid right lower lobe lung mass and subcarinal lymph node on PET scan. An EBUS FNA of a subcarinal lymph node and a transbronchial biopsy of the right lower lobe lung mass were performed. The air-dried DQ-stained smears from the EBUS FNA showed discohesive and cohesive cells with scattered multinucleated giant cells, in a lacy reticular purple-blue background [Figure 6a and 6b]. Some of the discohesive cells had dense cytoplasm with occasional long cytoplasmic tails (tadpole cells), while others appeared as stripped nuclei [Figure 6b]. The tumor cell nuclei were hyperchromatic with occasional nucleoli. The ROSE preliminary diagnosis was malignant neoplasm; however, it was uncertain as to what subtype of tumor was represented given the tigroid-like background. Cells with similar cytomorphology were seen on the Pap-stained alcohol fixed smears as well, without the lacy background material. The cell block and transbronchial biopsy showed similar histological features of tumor cells with eosinophilic cytoplasm in small nests with nuclear pleomorphism [Figure 6c]. Immunohistochemical and special stains performed on the cell block and transbronchial lung biopsy confirmed that the cells were positive for CK5/6, p63 and PAS [Figure 6d], and negative for TTF1. The final diagnosis for both the EBUS FNA of the subcarinal lymph node and the transbronchial biopsy was poorly differentiated squamous cell carcinoma.

The difficulty faced here was that the malignant cells showed squamous differentiation; however, the tigroid-like background raised the possibility of a mediastinal seminoma or dysgerminoma or other clear cell tumor. This case illustrates the difficulty that can occur when the cell population and background material appear to send us different clues, similar to the issue raised in the prior case (case 4). Thus, a thorough evaluation of the cytomorphology is important. For instance, in this case, the tumor cells appear to have dense cytoplasmic tails (tadpole cells; Figure 6b) and scattered cells with marked nuclear atypia [Figure 6a inset]. The tumor cells also lack the prominent nucleoli that are seen in seminomas and dysgerminomas. In addition, given that this specimen is from a female patient, the likelihood of a metastatic dysgerminoma or mediastinal germ cell tumor would be exceedingly rare. Thus, awareness of a tigroid-like background occurring in tumors other than germ cell tumors is important in EBUS FNA, particularly because metastatic carcinomas are a more common occurrence than mediastinal germ cell tumors and are treated differently.

The tigroid background in cytology was originally used to describe the lacy background material on air-dried smears stained with Romanowsky stains due to the striped look of the glycogen-rich cytoplasm after it is stripped from tumor cells. A tigroid-like background has also been described in aspiration cytology in tumors other than seminomas, and this usually involves glycogenated tumors, such as squamous cell carcinoma,[43] Ewings sarcoma,[44] and clear cell tumors.[45–47] There are also rare reports of this type of background being identified in adenomatoid tumors.[48] In addition, seminomas can be seen in the absence of a tigroid background,[49,50] and in one center’s experience, it was seen in less than 40% of seminomas on aspiration cytology.[51] In difficult cases, ancillary studies performed on the cytology material can help to subtype the type of tumor giving rise to the tigroid background. For example, seminomas and dysgerminomas will be positive for germ cell markers (PLAP, c-kit, Oct 3/4), squamous cell carcinomas will be positive for epithelial and squamous markers (cytokeratin, CK5/6, p63), and Ewing's sarcoma will be positive for CD99 and show the characteristic translocation involving the EWS gene by fluorescence in-situ hybridization. Table 2 summarizes the differential diagnosis for EBUS FNAs with a tigroid-like background.

## CONCLUSIONS

In conclusion, our study highlights some of the diagnostic difficulties encountered while evaluating EBUS FNAs at the time of ROSE. Many of the challenges that arise in these specimens can be attributed to the baseline cellularity of the aspirates (with lymphocytes and bronchial epithelial cells), the contamination from the needle traversing the bronchial wall (mucin and bronchial epithelial cells), the variety of entities that can occur with overlapping cytomorphologic features, and the rarity of published material on the cytomorphology of these specimens. Unlike most other specimens, the ROSE in EBUS FNA is crucial to determine if the patient will go directly for more invasive surgery (i.e., mediastinoscopy),[10] and therefore, there is not enough time for ancillary studies. Thus, the preliminary diagnosis is based on cytomorphology and clinical findings. Although ROSE may not always reach a definitive diagnosis, as seen in two of our cases and reported in the literature,[31] the immediate evaluation usually can give important preliminary information that can help to guide management.

In addition, the diagnostic challenges seen in EBUS-guided FNAs are somewhat different from other diagnostic modalities in the mediastinum and lung, such as CT-guided FNAs. To begin with, a CT-guided FNA that is nondiagnostic or negative for malignant cells, is usually quite hypocellular. Thus, any quantitative increase in cellularity is easy to see at the time of ROSE and usually indicates a pathologic process (i.e., infection, inflammation, neoplasia). In addition, aside from occasional mesothelial cells, soft tissue elements, and rarely hepatocytes (in right lower lobe FNAs), contamination is usually not a problem in CT-guided FNAs. However, in EBUS-guided FNAs, a nondiagnostic or negative specimen can be very cellular with numerous reactive bronchial cells (if nondiagnostic) and numerous lymphocytes (if diagnostic). This high cellularity creates a large number of diagnostic difficulties and makes these cases difficult to screen at the time of ROSE. Thus, the focus is not so much on the quantitative features, but more on the qualitative features and the need to determine if there are a sufficient number of the correct cells to indicate sampling of the target (i.e., do I have sufficient lymphocytes to indicate that the lymph node was adequately sampled?), and if there are malignant cells among all the benign and reactive cells (bronchial epithelial cells and lymphocytes). Thus, the approach to adequacy and diagnosis in EBUS cases is somewhat different from CT-guided FNA, and the cases illustrated in this report highlight some of the challenges in these specimens.

In conclusion, these cases highlight the importance of clinical history, cyto-histological correlation, communication with the performer of the FNA, and the need to thoroughly examine these cellular FNA specimens for atypical or malignant cells, in order to reach the most accurate diagnosis, to avoid important diagnostic pitfalls, and to ultimately maximize the concordance between preliminary and final diagnoses.

## COMPETING INTEREST STATEMENT BY ALL AUTHORS

No competing interest to declare by any of the authors.

## AUTHORSHIP STATEMENT BY ALL AUTHORS

Each author acknowledges that this final version was read and approved. All authors of this article declare that we qualify for authorship as defined by ICMJE http://www.icmje.org/#author . Each author has participated sufficiently in the work and take public responsibility for appropriate portions of the content of this article.

## EDITORIAL / PEER-REVIEW STATEMENT

To ensure integrity and highest quality of CytoJournal publications, the review process of this manuscript was conducted under a double blind model (authors are blinded for reviewers and reviewers are blinded for authors) through automatic online system.

## References

[1] Gonzalez-Stawinski GV, Lemaire A, Merchant F, O’Halloran E, Coleman RE, Harpole DH (2003). A comparative analysis of positron emission tomography and mediastinoscopy in staging nonsmall cell lung cancer. J Thorac Cardiovasc Surg.

[2] Graeter TP, Hellwig D, Hoffmann K, Ukena D, Kirsch CM, Schäfers HJ (2003). Mediastinal lymph node staging in suspected lung cancer: comparison of positron emission tomography with F-18-fluorodeoxyglucose and mediastinoscopy. Ann Thorac Surg.

[3] Kozower BD, Meyers BF, Reed CE, Jones DR, Decker PA, Putnam JB (2008). Does positron emission tomography prevent nontherapeutic pulmonary resections for clinical stage IA lung cancer?. Ann Thorac Surg.

[4] Kozower BD, Stukenborg GJ, Lau CL, Jones DR (2008). Measuring the quality of surgical outcomes in general thoracic surgery: should surgical volume be used to direct patient referrals?. Ann Thorac Surg.

[5] Gilbert S, Wilson DO, Christie NA, Pennathur A, Luketich JD, Landreneau RJ (2009). Endobronchial ultrasound as a diagnostic tool in patients with mediastinal lymphadenopathy. Ann Thorac Surg.

[6] Jacob-Ampuero MP, Haas AR, Ciocca V, Bibbo M (2008). Cytologic accuracy of samples obtained by endobronchial ultrasound-guided transbronchial needle aspiration at Thomas Jefferson University Hospital. Acta Cytol.

[7] Varela-Lema L, Fernández-Villar A, Ruano-Ravina A (2009). Effectiveness and safety of endobronchial ultrasound-transbronchial needle aspiration: a systematic review. Eur Respir J.

[8] Vincent BD, El-Bayoumi E, Hoffman B, Doelken P, DeRosimo J, Reed C (2008). Real-time endobronchial ultrasound-guided transbronchial lymph node aspiration. Ann Thorac Surg.

[9] Yasufuku K, Nakajima T, Motoori K, Sekine Y, Shibuya K, Hiroshima K (2006). Comparison of endobronchial ultrasound, positron emission tomography, and CT for lymph node staging of lung cancer. Chest.

[10] Cameron SE, Andrade RS, Pambuccian SE (2010). Endobronchial ultrasound-guided transbronchial needle aspiration cytology: a state of the art review. Cytopathology.

[11] Herth F, Becker HD, Ernst A (2004). Conventional vs endobronchial ultrasound-guided transbronchial needle aspiration. Chest.

[12] Yasufuku K, Chiyo M, Sekine Y, Chhajed PN, Shibuya K, Iizasa T (2004). Real-time endobronchial ultrasound-guided transbronchial needle aspiration of mediastinal and hilar lymph nodes. Chest.

[13] Alsharif M, Andrade RS, Groth SS, Stelow EB, Pambuccian SE (2008). Endobronchial ultrasound-guided transbronchial fine-needle aspiration: the University of Minnesota experience, with emphasis on usefulness, adequacy assessment, and diagnostic difficulties. Am J Clin Pathol.

[14] Feller-Kopman D, Yung RC, Burroughs F, Li QK (2009). Cytology of endobronchial ultrasound-guided transbronchial needle aspiration: a retrospective study with histology correlation. Cancer Cytopathol.

[15] Crapanzano JP, Zakowski MF (2001). Diagnostic dilemmas in pulmonary cytology. Cancer.

[16] Linder J (1997). Errors and pitfalls in lung and pleural cytology. Monogr Pathol.

[17] Thivolet-Béjui F (1997). Cytological pitfalls in bronchopulmonary tumors. Diagn Cytopathol.

[18] Jin N, Nguyen C, Shuja S, Makary R, Wolfson D (2009). Cytomorphology of primary signet ring-cell carcinoma of the lung. Diagn Cytopathol.

[19] Laucirica R, Ostrowski ML (2007). Cytology of nonneoplastic occupational and environmental diseases of the lung and pleura. Arch Pathol Lab Med.

[20] Li C, Kim S, Lai JF, Hyung WJ, Choi WH, Choi SH (2007). Advanced gastric carcinoma with signet ring cell histology. Oncology.

[21] Bal A, Kumar Y, Das A, Bhatia A (2008). Signet ring cell plasmacytoma: a rare morphological variant. Pathology.

[22] Magro CM, Crowson AN, Mihm MC (2006). Unusual variants of malignant melanoma. Mod Pathol.

[23] Cook DS, Attanoos RL, Jalloh SS, Gibbs AR (2000). ‘Mucin-positive’ epithelial mesothelioma of the peritoneum: an unusual diagnostic pitfall. Histopathology.

[24] Ordóñez NG (2005). Mesothelioma with clear cell features: an ultrastructural and immunohistochemical study of 20 cases. Hum Pathol.

[25] Ulbright TM, Young RH (2008). Seminoma with conspicuous signet ring cells: a rare, previously uncharacterized morphologic variant. Am J Surg Pathol.

[26] Tozbikian G, Shen R, Suster S (2008). Signet ring cell gastric schwannoma: report of a new distinctive morphological variant. Ann Diagn Pathol.

[27] Eltoum IA, Tabbara S (1998). Intraoperative Cytologic Diagnosis of Granulomas: a retrospective study of 156 cases. Diagn Cytopathol.

[28] Nagarjun Rao R, Moran CA, Suster S (2010). Histiocytic disorders of the lung. Adv Anat Pathol.

[29] Zardawi IM (1999). Renal fine needle aspiration cytology. Acta Cytol.

[30] Truong LD, Todd TD, Dhurandhar B, Ramzy I (1999). Fine-needle aspiration of renal masses in adults: analysis of results and diagnostic problems in 108 cases. Diagn Cytopathol.

[31] Woon C, Bardales RH, Stanley MW, Stelow EB (2006). Rapid assessment of fine needle aspiration and the final diagnosis- how often and why the diagnoses are changed. Cytojournal.

[32] Wiatrowska BA, Krol J, Zakowski MF (2001). Large-cell neuroendocrine carcinoma of the lung: proposed criteria for cytologic diagnosis. Diagn Cytopathol.

[33] Dresler CM, Ritter JH, Patterson GA, Ross E, Bailey MS, Wick MR (1997). Clinical-pathologic analysis of 40 patients with LCNEC of the lung. Ann Thorac Surg.

[34] Sundaresan V, Reeve JG, Stenning S, Stewart S, Bleehen NM (1991). Neuroendocrine differentiation and clinical behavior in nonsmall cell lung tumours. Br J Cancer.

[35] Hammond ME, Sause WT (1985). Large cell neuroendocrine tumors of the lung.Clinical significance and histopathologic definition. Cancer.

[36] Rossi G, Cavazza A, Marchioni A, Longo L, Migaldi M, Sartori G (2005). Role of chemotherapy and the receptor tyrosine kinases KIT, PDGFR-alpha, PDGFR-beta, and Met in large-cell neuroendocrine caecinoma of the lung. J Clin Oncol.

[37] Dugan JM (1995). Cytologic diagnosis of basal cell (basaloid) carcinoma of the lung.A report of two cases. Acta Cytol.

[38] Khalbuss WE, Fischer G, Tutuncuoglu SO (2007). Cytomorphology of basaloid (basal cell) carcinoma of the lung. Acta Cytol.

[39] Sturm N, Lantuéjoul S, Laverrière MH, Papotti M, Brichon PY, Brambilla C (2001). Thyroid transcription factor 1 and cytokeratins 1, 5, 10, 14 (34betaE12) expression in basaloid and large-cell neuroendocrine carcinomas of the lung. Hum Pathol.

[40] Pelosi G, Rodriguez J, Viale G, Rosai J (2005). Typical and atypical pulmonary carcinoid tumor overdiagnosed as small-cell carcinoma on biopsy specimens. Am J Surg Pathol.

[41] Nicholson SA, Ryan MR (2000). A review of cytologic findings in neuroendocrine carcinomas including carcinoid tumors with histologic correlation. Cancer.

[42] Chhieng DC (2008). Fine-needle aspiration cytology of pulmonary mucinous cystadenocarcinoma. Diagn Cytopathol.

[43] Dusenbery D (1997). Aspiration cytology of glycogen-rich squamous-cell carcinoma: significance of a tigroid smear background. Acta Cytol.

[44] Miralles TG, Gosalbez F, Menéndez P, Astudillo A, Torre C, Buesa J (1986). Fine needle aspiration cytology of soft tissue lesions. Acta Cytol.

[45] Hirokawa M, Shimizu M, Nakamura E, Kanahara T, Yamauchi H, Fujiwara K (2000). Basement membrane material and tigroid background in a fine-needle aspirate of clear cell adenocarcinoma of the cervix. Acta Cytol.

[46] Rau AR, Kini H, Verghese R (2006). Tigroid background in fine-needle aspiration cytology of clear cell sarcoma. Diagn Cytopathol.

[47] Khunamornpong S, Thorner PS, Suprasert P, Siriaunkgul S (2005). Clear-cell adenocarcinoma of the female genital tract: presence of hyaline stroma and tigroid background in various types of cytologic specimens. Diagn Cytopathol.

[48] Monappa V, Rao AC, Krishnanand G, Mathew M, Garg S (2009). Adenomatoid tumor of tunica albuginea mimicking seminoma on fine needle aspiration cytology: a case report. Acta Cytol.

[49] Caraway NP, Fanning CV, Amato RJ, Sneige N (1995). Fine-needle aspiration cytology of seminoma: a review of 16 cases. Diagn Cytopathol.

[50] Kwon MS (2005). Aspiration cytology of mediastinal seminoma: report of a case with emphasis on the diagnostic role of aspiration cytology, cell block and immunocytochemistry. Acta Cytol.

[51] Gupta R, Mathur SR, Arora VK, Sharma SG (2008). Cytologic features of extragonadal germ cell tumors: a study of 88 cases with aspiration cytology. Cancer.

